# Significance of pulse pressure variability in predicting functional outcome in acute ischemic stroke: a retrospective, single-center, observational cohort study

**DOI:** 10.1038/s41598-023-30648-2

**Published:** 2023-03-03

**Authors:** Maria Kamieniarz-Mędrygał, Radosław Kaźmierski

**Affiliations:** 1 Department of Neurology, S. T. Dąbrowski Hospital in Puszczykowo, Kraszewskiego Str. 11, 62-041 Puszczykowo, Poland; 2 Poznan University of Medical Sciences, Poznan, Poland; 3 Department of Neurology, Collegium Medicum, University of Zielona Gora, Zielona Gora, Poland; 4 Department of Neurology, Poznan University of Medical Sciences, Poznan, Poland

**Keywords:** Stroke, Hypertension

## Abstract

This study aimed to determine the association between pulse pressure variability (PPV) and short- and long-term outcomes of acute ischemic stroke (AIS) patients. We studied 203 tertiary stroke center patients with AIS. PPV during 72 h after admission was analyzed using different variability parameters including standard deviation (SD). Patients’ outcome was assessed after 30 and 90 days post-stroke with modified Rankin Scale. The association between PPV and outcome was investigated using logistic regression analysis with adjustment for potential confounders. The predictive significance of PPV parameters was determined using area under the curve (AUC) of receiver operating characteristics. In the unadjusted logistic regression analysis, all PPV indicators were independently associated with unfavorable outcome at 30 days (i.a. Odds ratio (OR) = 4.817, 95%CI 2.283–10.162 per 10 mmHg increase in SD, p = 0.000) and 90 days (i.a. OR = 4.248, 95%CI 2.044–8.831 per 10 mmHg increase in SD, p = 0.000). After adjustment for confounders, ORs for all PPV indicators remained statistically significant. On the basis of AUC values, all PPV parameters were found relevant outcome predictors (p < 0.01). In conclusion, elevated PPV during first 72 h after admission due to AIS is associated with unfavorable outcome at 30 and 90 days, independent of mean blood pressure levels.

## Introduction

Acute hypertensive response occurs frequently in patients during ischemic stroke, but the pathophysiology of that phenomenon remains unknown^1^. Furthermore, it is not clear how to properly control blood pressure (BP) in hyperacute ischemic stroke^2^.Current guidelines suggest setting only the upper threshold of BP^3^. This threshold is different, depending whether the patient is undergoing reperfusion therapy such as intravenous thrombolysis (IVT) or mechanical thrombectomy (MT) or not undergoing such treatment^3^. Therapeutical intervention for BP control is only recommended if the upper BP threshold is exceeded, or in case some specific comorbidity (e.g. acute heart failure, acute coronary event, aorta dissection, preeclampsia) makes it necessary^4^. Current guidelines lack recommendations regarding the lower BP threshold as well as acceptable BP fluctuations in the acute period of stroke. In absence of randomized trials, recommendations are based on observational and retrospective studies and, as a consequence, their strength is weak^5,6^. Recent studies have not shown any benefit of aggressive BP reduction in hyperacute period^7,8^. By contrast, studies have proved the “U” shaped relationship between BP levels and worse patients outcome and death^9,10^.

Beside absolute BP levels, also blood pressure variability (BPV) turned out to be associated with unfavorable outcome^11,12^. BPV might be responsible for hypoperfusion and hyperperfusion of vulnerable ischemic penumbra, resulting in either enlargement of the ischemic area and cerebral oedema or hemorrhagic transformation. Currently, there is no unified research methodology for measuring BPV, precluding meta-analysis^13,14^. Most studies estimate BP fluctuations by systolic blood pressure (SBP) and mean arterial pressure (MAP)^15^. Diastolic blood pressure (DBP) is less frequently used, and only a few studies employed pulse pressure (PP). Independently of which BP component was the focus, studies up to date have typically used only a single BPV statistic parameter, usually standard deviation (SD), and only few publications took into account several different parameters^15^.

Elevated PP is associated with poor cardiovascular prognosis^16^. It was also demonstrated to be associated with post-stroke mortality and stroke recurrence^17–19^. Pulse pressure is a pulsatile and dynamic part of BP so it may better describe BP variability. However, only few studies addressed the role of PP variability (PPV) on stroke outcome and they only used single parameters^20,21^. Nevertheless they have found an association between high PPV and unfavorable clinical outcome in patients with AIS who underwent either IVT^20^ as well as prognostic significance of PPV for AIS patients treated with MT^21^. The retrospective study in a group of patients not qualified to the thrombolytic treatment revealed also that PPV provided a prime predictor of bad outcome^22^. Hence, the aim of this study was to determine the relationship between PPV and stroke short- and long-term outcome using a variety of statistic parameters.

## Methods

### Study subjects and data collection

**Figure 1:**
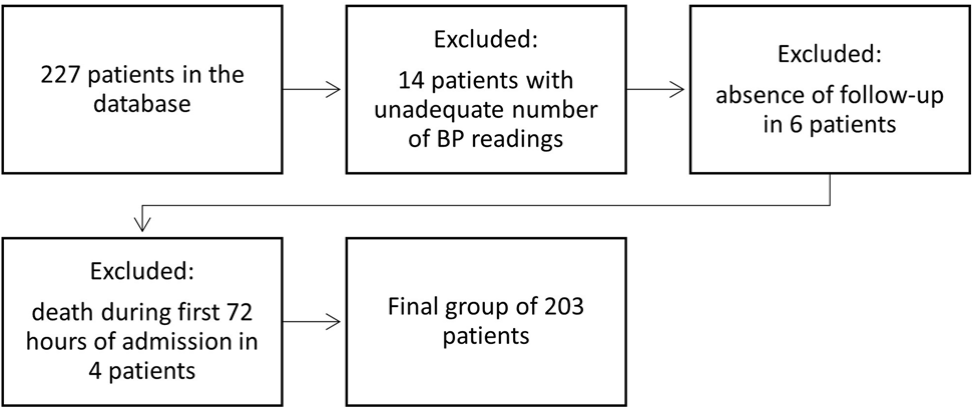
Flowchart showing categories of patients excluded from the study.

The study has a retrospective character and is based on an electronic database registered in our university stroke unit. The patients study group was collected in 2009–2011. Patients included in the study cohort were admitted to the hospital within 36 h from the onset of stroke symptoms. At admission, all patients were evaluated using National Institutes of Health Stroke Scale (NIHSS) and Glasgow Coma Scale^23^. At the same time, demographic data, comorbid conditions, history of previous cardiovascular diseases and baseline measures were collected. Further on, all patients underwent non-enhanced head computer tomography (CT) or magnetic resonance imaging (MRI) at admission and within 2–5 days after stroke onset. Patients were treated employing medication and care according to current national stroke guidelines^24^. From 227 patients collected in the database, 24 patients were excluded from further analysis because of insufficient data (lack of adequate number of BP measures in 14 patients, absence of follow-up visit in 6 patients) or death during the period of first 72 h post-stroke (4 patients). The flowchart in Fig. 1 shows the exclusions, which were mostly due to interruptions in successive four-hour-interval BP readings needed for the variability estimates. Patients’ written consent on participation in the study and collection of personal data was obtained during the time of hospitalization. Ethical approval for this study was issued by the Chairman of the *Committee* on Bioethics, Poznan University of Medical Sciences (decision from 15.04.2021). This study was completed in accordance with the Helsinki Declaration as revised in 2013.

### BPV parameters

Blood pressure values were taken in the supine position in the non-paretic arm by a trained nurse using the Ultraview SL2600 monitoring system (Spacelabs Medical Inc., USA), which meets and exceeds the American National Standards Institute/Association for the Advancement of Medical Instrumentation (ANSI/AAMI) standard SP-10. In further analysis we used blood pressure measurements recorded in 4-h intervals from midnight after admission through next 72 h of hospitalization. PP was calculated as a difference between systolic and diastolic blood pressure (SBP—DBP). PPV was investigated using varied parameters, all employed in former studies^22,25^. The following formulae, where *x*_i_ and $\overline{x }$ stand for a single PP reading and the mean value of all readings, respectively, were exploited: standard deviation (SD, $SD= \sqrt{1/(n-1){\sum_{i=1}^{n}({x}_{i}-\overline{x })}^{2}}$), coefficient of variation (CV, CV = 100xSD/$\overline{x }$), successive variation index (SV, $SV=\sqrt{1/(n-1)\sum_{i=1}^{n-1}{({x}_{i+1}-{x}_{i})}^{2}}$), average real variability (ARV, $ARV=1/(n-1){\sum }_{i=1}^{n-1}\left|{x}_{i+1}-{x}_{i}\right|$), difference maximum-minimum (DMM; difference between maximum and minimum PP value recorded) and maximal successive change (MSC; the highest difference between successive readings).

### Functional outcome

The clinical outcome was assessed by trained neurologists at 30-days (short-term) and 90-days (long-term) follow-up, using the modified Rankin Scale (mRS). The functional assessment was preceded by an intraobserver reliability check comprising independent neurologists involved in prior studies^26^. Data on the degree of disability and independence in daily activities was gathered during a visit or by a telephone survey. It was obtained directly from the patients whenever possible, and otherwise from their caregivers. The functional outcome was dichotomized based on mRS score, unfavorable outcome was defined as mRS score ≥ 3 (dependance/death) while favorable outcome as mRS ≤ 2. This dichotomized division was used for the analyses employing the Mann–Whitney U test and in Model 1, 3 and 4 of logistic regression. In Model 2 of logistic regression, a ‘severity-adjusted’ outcome relying on admission NIHSS score, was employed^27^. In this case, the outcome was considered unfavorable when mRS score was 2–6 and NIHSS was ≤ 7, or mRS was 3–6 and NIHSS was between 8 and 14, or mRS was 4–6 and NIHSS was > 14. In other cases the outcome was considered favorable.

### Statistical analysis

All data analysis was performed using Statistica 13.3 package (Tibco). All the items available in the database and relevant to the study were taken into account without prior sample size calculations. However, in our analysis the sample size is higher by more than one order of magnitude with respect to number of variables. Quantitative data were expressed as mean ± SD or median (interquartile range). Categorical variables were described as numbers (percentage). *P* values < 0.05 were considered statistically significant. To determine the relationship between PPV parameters and functional outcome in the groups considered, i.e. with/without thrombolytic treatment and the entire cohort, Spearman rank correlation test was used as a first approach. To assess the relationship, statistical significance of the correlation coefficients was invoked, considering moderate values of ρ_S_ < 0.4. Similar moderate correlations of BPV indicators were reported before in stroke studies^28^. The statistical significance of differences between the groups of patients with/without thrombolytic treatment were evaluated with the Mann–Whitney U test. The association between PPV parameters and unfavorable outcome was examined with multivariable logistic regression analysis. The Odds ratios (OR) and 95% confidence intervals (CIs) were calculated per 10 mmHg increase in PPV parameter, as recommended in prior systematic review^15^. The predictive significance of PPV parameters was determined using receiver operating characteristics (ROC) curve analysis. The area under the curve (AUC) values, Youden’s Index and cut-off points were reported.

### Informed consent

Written informed consent was obtained from all subjects before the study (at the time of original data collection).

## Results

203 patients were included in the study, out of which 48 (20%) were subject to thrombolytic treatment. None of the patients included underwent MT therapy. From the whole cohort, 82 patients achieved unfavorable outcome (mRS ≥ 3) at 30-days and 79 at 90-days after stroke. Patients were more likely to be dependent or dead at 30 days if they were female, were older or had higher admission NIHSS (left side of Table 1).

**Table 1:** Baseline characteristics of two study cohorts for 30-days and 90-days post-stroke outcome.

	mRs < 3 (n = 121)	mRs ≥ 3 (n = 82)	U test *p* value	mRs < 3 (n = 124)	mRs ≥ 3 (n = 79)	U test *p* value
	30-days outcome			90-days outcome		
Age (years: mean ± SD)	64 ± 13	73 ± 12	**0.000**	64 ± 13	73 ± 12	**0.000**
Female	49 (40.5%)	47 (57.3%)	**0.019**	50 (40.3%)	46 (58.2%)	**0.013**
Past medical history						
Diabetes	24 (19.8%)	18 (22%)	**0.717**	23 (18.6%)	19 (24.1%)	0.347
Hypertension	76 (62.8%)	58 (70.7%)	0.244	79 (63.7%)	55 (69.6%)	0.388
Atrial fibrillation	24 (19.8%)	25 (30.5%)	0.083	25 (20.2%)	24 (30.4%)	0.098
Heart failure	9 (7.4%)	10 (12.2%)	0.256	10 (8.1%)	9 (11.39%)	0.200
Myocardial infarction	13 (10.7%)	15 (18.3%)	0.127	12 (9.7%)	16 (20.3%)	**0.034**
Stroke	22 (18.2%)	19 (23.2%)	0.387	21 (16.9%)	20 (25.3%)	0.149
Smoking	25 (20.7%)	13 (15.9%)	0.295	26 (21%)	12 (15.2%)	0.228
Coronary heart disease	29 (24%)	23 (19%)	0.515	27 (21.8%)	25 (31.7%)	0.117
Clinical features						
Admission NIHSS (median, IQR)	5 (3–7)	15 (7–19)	**0.000**	5 (3–7)	14 (7–19)	**0.000**
Thrombolysed	25 (20.7%)	13 (15.9%)	0.391	25 (20.2%)	13 (16.5%)	0.512
Admission SBP (mmHg: mean ± SD)	154 ± 23	155 ± 29	0.769	153 ± 24	156 ± 28	0.68
Admisssion DBP (mmHg: mean ± SD)	91 ± 15	91 ± 21	0.622	91 ± 15	90 ± 21	0.394
Admission PP (mmHg: mean ± SD)	63 ± 20	65 ± 22	0.420	63 ± 19	66 ± 22	0.208

DBP: diastolic blood pressure; mRS: modified Rankin scale; NIHSS: National Institutes of Health Stroke Scale; PP: Pulse Pressure; SD: standard deviation; SBP: systolic blood pressure.

Significant values are in bold.

This also applied to the group with unfavorable outcome at 90 days (right side of Table 1). Additionally, there was no association between admission BP values and stroke short- and long term outcome (Table 1).

The relationship between PPV and 30 and 90 days outcome was analyzed in the thrombolysis and non-thrombolysis subgroups as well as in the whole cohort, using the Spearman rank correlation test (Table 2). All PPV indices were significantly associated with mRS score after 30- and 90-days (p < 0.05), with the exception of CV in the thrombolysis group. Interestingly, higher Spearman ρ values were observed in patients after recanalization therapy (Spearman ρ = 0.455 for MSC after 90 days outcome). According to the Mann–Whitney U test, there was no difference between mean PPV values in the groups with- or without thrombolytic treatment (p > 0.05, right side of Table 2). Based on this result, further analyses were performed in the whole cohort (n = 203). We observed that correlations between mean PP values and mRS scores were only relevant in the group with thrombolytic treatment (Table 2).

**Table 2:** Association between PPV parameters measured during 72 h after admission and outcome determined by mRS at 30- and 90-days post-stroke period (in bold if p < 0.05).

PP parameter	Thrombolysis therapy n = 38			Non-thrombolysis therapy n = 165			All n = 203		
	72 h	30 d.o.*	90 d.o.†	72 h	30 d.o.*	90 d.o.†	U test‡	30 d.o.*	90 d.o.†
	mean ± SD	ρ_S_	ρ_S_	mean ± SD	ρ_S_	ρ_S_	p	ρ_S_	ρ_S_
PP CV	22.3 ± 7.1	0.164	0.136	22.4 ± 7.8	**0.272**	**0.253**	0.955	**0.248**	**0.236**
PP SV	15.4 ± 4.1	**0.359**	**0.405**	16.3 ± 6.3	**0.288**	**0.252**	0.636	**0.295**	**0.277**
PP MSC	33.6 ± 12.0	**0.387**	**0.455**	35.8 ± 16.4	**0.262**	**0.241**	0.563	**0.287**	**0.285**
PP DMM	43.8 ± 14.4	**0.327**	**0.315**	47.1 ± 17.3	**0.310**	**0.290**	0.267	**0.321**	**0.307**
PP ARV	12.2 ± 3.0	**0.341**	**0.369**	12.7 ± 4.8	**0.279**	**0.251**	0.155	**0.278**	**0.262**
PP SD	11.4 ± 3.3	**0.329**	**0.295**	14.4 ± 5.4	**0.307**	**0.272**	0.733	**0.319**	**0.288**
PP mean	54.5 ± 15.5	**0.326**	**0.362**	57.9 ± 13.7	0.046	0.026	0.103	0.123	0.098

*30 d.o. = 30 days outcome; † 90 d.o. = 90 days outcome; ‡U—the test compares PP parameters between the groups with and without thrombolysis.

ARV: average real variability; CV: coefficient of variation; DMM: difference maximum-minimum; MSC: maximal successive change; PP: pulse pressure; ρ_S_: Spearman ρ; SD: standard deviation; SV: successive variation.

The next step of the study was the logistic regression analysis for short- and long-term outcome. The unadjusted model demonstrated that all PPV indices were associated with risk of unfavorable outcome at 30 and 90 days after AIS (Table 3, Model 1). In Model 2, all PPV indices were adjusted for their mean values and the application of recombined tissue plasminogen activator (rtPA). The PPV indices were independently associated with poor outcome at 30 days (OR = 4.457, 95% CI 1.961–10.132 per 10 mmHg increase in SD, p = 0.000; OR = 1.473, 95% CI 1.190–1.823 for 10 mmHg increase in MSC, p = 0.000) and 90 days (OR = 3.666, 95% CI 1.648–8.156 per 10 mmHg increase in SD, p = 0.001; OR 1.439, 95% CI 1.167–1.774 per 10 mmHg increase in MSC, p = 0.001). Very similar odds ratios (OR) were found for PPV indices when the ‘adjusted outcome’ was employed, which took into consideration both mRS and baseline NIHSS scores, as mentioned in the methods (Model 3). Model 4 was an extension of Model 2 adjusting for age, gender and history of myocardial infarction. We conclude that ORs for all PPV indicators remain statistically significant, although their values steadily decrease, passing from Model 1 to Model 4. The exception are PP ARV and PP SD indices, for which the ORs are not statistically significant in Model 4 as far as the long-term outcome is concerned (right-bottom corner of Table 3).

**Table 3:** Multivariate logistic regression analysis showing the PPV association with unfavorable outcome at 30- and 90-days post-stroke period.

PP parameter		Model 1		Model 2 + rtPA + mean PP			Model 3 Severity adjusted outcome + rtPA + mean PP			Model 4 Model 2 + age + gender + MI		
	p	OR	95% CI	p	OR	95% CI	p	OR	95% CI	p	OR	95% CI
Outcome after 30 days												
PP CV	0.007	1.742	1.161–2.615	0.001	2.193	1.400–3.437	0.004	1.897	1.229–2.927	0.017	1.768	1.107–2.823
PP SV	0.000	2.925	1.712–4.999	0.001	2.687	1.522–4.745	0.004	2.277	1.301–3.983	0.016	2.072	1.145–3.747
PP MSC	0.000	1.522	1.241–1.867	0.000	1.473	1.190–1.823	0.002	1.388	1.123–1.715	0.010	1.335	1.071–1.664
PP DMM	0.000	1.476	1.220–1.784	0.001	1.436	1.171–1.760	0.000	1.460	1.184–1.802	0.012	1.312	1.063–1.619
PP ARV	0.000	3.855	1.910–7.780	0.001	3.420	1.620–7.217	0.014	2.472	1.200–5.094	0.021	2.518	1.152–5.502
PP SD	0.000	4.817	2.283–10.162	0.000	4.457	1.961–10.132	0.002	3.651	1.621–8.221	0.012	2.995	1.272–7.053
Outcome after 90 days												
PP CV	0.01	1.646	1.104–2.453	0.001	2.102	1.348–3.278	0.023	1.612	1.067–2.435	0.047	1.607	1.007–2.565
PP SV	0.000	2.861	1.678–4.878	0.001	2.548	1.451–4.474	0.013	1.990	1.159–3.416	0.040	1.861	1.030–3.364
PP MSC	0.000	1.502	1.227–1.838	0.001	1.439	1.167–1.774	0.007	1.323	1.080–1.620	0.027	1.282	1.029–1.596
PP DMM	0.000	1.454	1.205–1.755	0.001	1.397	1.143–1.707	0.00	1.333	1.095–1.622	0.035	1.253	1.015–1.545
PP ARV	0.000	3.733	1.854–7.519	0.002	3.172	1.511–6.659	0.023	2.286	1.122–4.658	0.051	2.181	0.997–4.772
PP SD	0.000	4.248	2.044–8.831	0.001	3.666	1.648–8.156	0.018	2.520	1.174–5.413	0.064	2.212	0.953–5.131

Model 1 unadjusted logistic regression, Model 2 adjusted to rtPA application and mean PP; Model 3 severity adjusted outcome adjusted to rtPA and mean PP; Model 4 adjusted to rtPA application, mean PP, age, gender and history of myocardial infarction.

ARV: average real variability; CI: confidence interval; CV: coefficient of variation; DMM: difference maximum-minimum; MI: myocardial infarction; MSC: maximal successive change; OR: Odd’s ratio; PP: pulse pressure; PPV: pulse pressure variability; rtPA: recombined tissue plasminogen activator; SD: standard deviation; SV: successive variation.

The quality and usefulness of PPV indices as predictors of unfavorable outcome was also examined by the ROC curve analysis (Table 4).

**Table 4:** The results of the ROC curve analysis determining predictive power of PPV indicators of unfavorable outcome at 30- and 90-days period after stroke onset.

PP parameter	30 days outcome					90 days outcome						
	AUC	AUC CI	C-f*	YI†	p	AUC	AUC CI		C-f*	YI†		p
PP CV	0.619	0.538–0.699	22	0.25	0.004	0.608	0.526–0.689		22	0.29		0.01
PP SV	0.659	0.582–0.736	16	0.27	0.000	0.659	0.581–0.738		16	0.29		0.000
PP MSC	0.661	0.584–0.737	39	0.26	0.000	0.659	0.58–0.737		39	0.26		0.000
PP DMM	0.669	0.593–0.744	45	0.26	0.000	0.669	0.591–0.746		51	0.3		0.000
PP ARV	0.649	0.571–0.726	14	0.25	0.000	0.648	0.568–0.727		14	0.28		0.000
PP SD	0.675	0.598–0.751	15	0.29	0.000	0.664	0.585–0.742		15	0.3		0.000

*C-f = Cut-off point.

^†^YI = Youden’s Index.

ARV: average real variability; AUC: area under the curve; CI: confidence interval; CV: coefficient of variation; DMM: difference maximum-minimum; MSC: maximal successive change; PP: pulse pressure; PPV: pulse pressure variability; SD: standard deviation; SV: successive variation.

**Figure 2:**
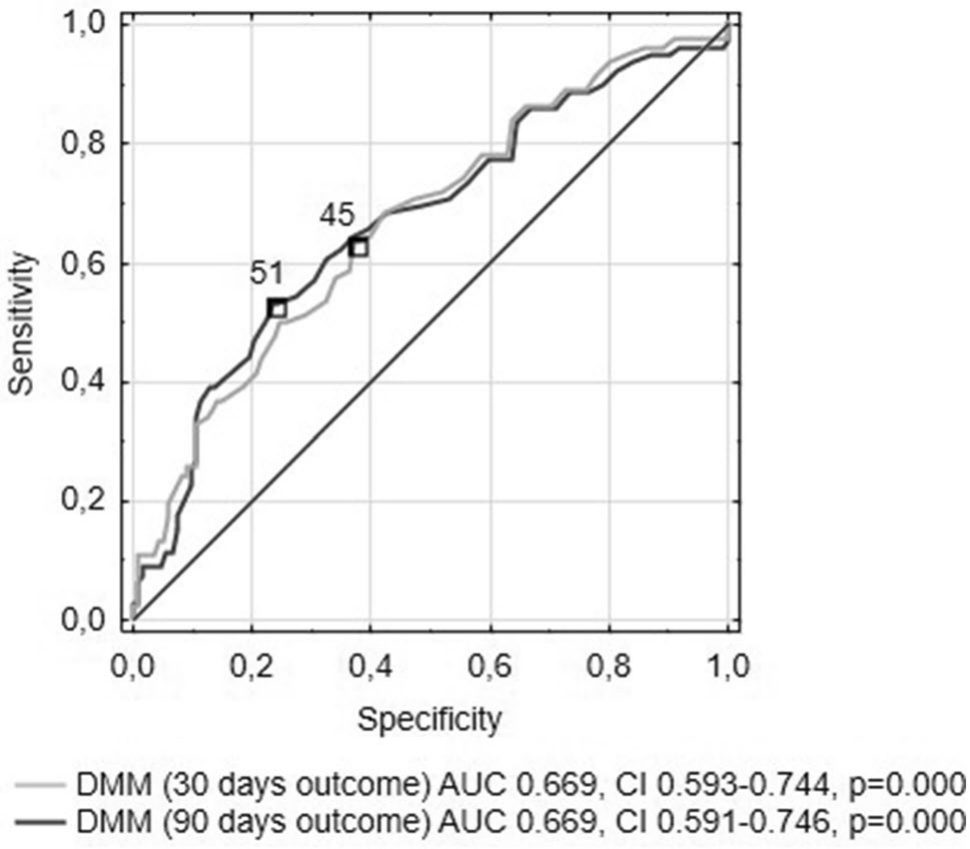
ROC analysis of PP DMM and 30- and 90-days unfavorable outcome. AUC area under the curve, CI confidence interval, DMM difference maximum-minimum, PP pulse pressure, ROC receiver operating characteristic.

On the basis of AUC values, all PPV parameters were found relevant outcome predictors (p < 0.01). The highest values of AUC amounts to 0.669 for PP DMM index both at 30- and 90-days post-stroke (Fig. 2). We note, however, that only in this case the cut-off points are different for these two periods.

## Discussion

Our study demonstrates that increased PPV in the acute ischemic stroke phase was associated with both short- and long-term unfavorable outcome. Every 10 mmHg increase in the 72-h PPV indices leads to a higher likelihood of unfavorable outcome at 30- and 90-days post-stroke. The association of PPV with functional outcome held up even after adjustment for mean PP values, thrombolysis treatment and other baseline characteristics. We found that all PPV indices were valuable outcome predictors. Our study shows that DMM and MSC indices, which are very easy to compute, are as reliable as other, more complex indicators (CV, SV, SD, ARV). Therefore, DMM and MSC seem the most convenient in clinical practice.

Previous studies evaluated BPV most frequently with the use of SBP and MAP components and demonstrated the association of BPV with poor outcome 90 days after AIS^11,12,29–32^. This association was more evident in studies involving patients after reperfusion therapies^12,31,32^. Some studies found that BPV is associated with the post-stroke outcome only in those patients, who underwent thrombolysis treatment and who did not achieve vessel recanalization, based on imaging examination^30,33^. This led to the view that in patients with successful recanalization increased BPV is less of a concern. However, as opposed to MT, in IVT therapy the exact time of recanalization is unknown. Observational studies examining the impact of BPV in patients treated with MT demonstrated a general significant relationship with poor outcome or death, which was not limited to non-recanalized patients^12,31^.

The role of PPV in clinical practice is currently underestimated. Up to date only few studies examined the relationship between PPV and stroke outcome^20–22,25^. Our results are consistent with other recent reports. Katsanos et al. conducted a study on a group of thrombolysis-eligible patients and found that increased PPV was independently associated with both short- (24-h) and long-term (90 days) outcome^20^. Every 5 mmHg-increase in the 24-h PP SD was independently associated with a 36% decrease in the likelihood of 90-day independent functional outcome. We found the association between PPV and poor outcome in the whole cohort of patients, i.e. with and without thrombolysis treatment. 10 mmHg increase in PP SD was significantly associated with unfavorable outcome at 90 days (OR_adjusted_ = 3.666, 95% CI 1.648–8.156, p = 0.001). Notably, higher Spearman correlation ρ’s were obtained here in the group with thrombolytic treatment (ρ = 0.455 for PP MSC after 90 days outcome). Because of insufficient amount of patients with thrombolysis, we could not include this group in the logistic regression analysis. Another study concerning PPV was carried out on patients with large vessel occlusion stroke treated with MT^25^. In that investigation, PPV 24 h after IAT was associated with poor 3-month outcome and PPV indices had an excellent ability to predict unfavorable outcome (AUC 0.924 for SD). For comparison, in our study, the corresponding results for the whole cohort were lower (AUC 0.664 for SD), but high enough to provide predictive significance (p = 0.000). In both studies, DMM emerged as an equally reliable outcome predictor as more complex indices (CV, SV, SD).

It is worth emphasizing that the significance of PP as a predictor of cardiovascular risk is well established^16,34–36^. The relationship between PP and cerebrovascular incidents was less investigated. Lee et al. found that PP in the acute period of stroke had a nonlinear, J-shaped relation with major vascular events, or stroke recurrence^19^. Notably, the predictive power of PP was stronger than that of other commonly used BP parameters (SBP, MAP). Another study demonstrated a non-linear reverse J-curve association between the admission PP level and 3-month post-stroke functional outcomes^37^. By contrast, we have not found any connection between admission PP values and post-stroke outcome after 30 and 90 days.

While MAP is defined as an average blood pressure in aorta and its major branches during the cardiac cycle and it is nearly constant along the arterial tree, PP is considered as a pulsatile component of BP. MAP and PP are dependent variables, though different PP values may occur for a given MAP^38^. It is observed that PP increases markedly with age^39^. Among the causes affecting PP raise, in young individuals stroke volume and ventricular ejection is dominant, whereas in elderly, PP is mainly affected by a reduction in visco-elastic properties of arterial wall and the timing of wave reflection^38,40^. Hence, PP is commonly taken as a marker of arterial stiffness. Arterial stiffness was reported to be associated with resistance in cerebral circulation in elderly^41^. It is also suspected to lead to the impairment of the collateral circulation and therefore, to decrease the benefit of recanalization therapies in acute ischemic stroke. Thus future trials investigating the association between PPV and collateral circulation in acute stroke patients are highly needed. Systolic BPV was found to be associated with 90-days post-stroke outcome in patients with poor collateral status, but the data concerning PPV are lacking^42,43^.

In our study group, risk factors of achieving worse clinical outcome or death were (1) female sex and (2) previous myocardial infarction. We used those factors as confounders in our logistic regression analysis. The association between BPV and cardiovascular events is well documented. Increased long-term BPV is significantly associated with coronary heart disease incidents and cardiovascular mortality, independent of mean BP^44,45^. Greater short-term BPV after acute coronary syndromes is a predictor of major adverse cardiac events^46,47^. The impact of sex on the magnitude of BPV and further, on ischemic stroke outcome has not been studied to the best of our knowledge. Women tend to have higher SBP at the time of presentation with AIS and are more likely to have premorbid hypertension^48^. The underlying mechanism for the observed sex differences is not clear, however it has been proposed that female steroid hormones and autonomic dysregulation after menopause are likely to play a role^49,50^. There is a great discrepancy in stroke outcome, with women having more severe strokes, less favorable prognoses and greater incidence of death^51,52^. Therefore we cannot exclude the possibility that our data may have been influenced by sex differences. In future trials it would be interesting to systematically study the sex disparities in BPV and their association with stroke outcome.

Our study had several limitations. It was a retrospective analysis of a prospective single-center stroke database, which might lead to selection bias and limits the generalizability of the results. The sample size is relatively small in relation to prevalence of the stroke incidents and we have used BP measurements in 4-h intervals, which is larger than suggested in recent literature^13^. The database does not contain the results of imaging techniques (CT, MRI) so that we could not consider secondary outcomes such as symptomatic intracerebral hemorrhage or cerebral infarct volume. In addition, it does not provide information about implemented drug treatments, which is a factor possibly affecting BP values. Importantly, we used standard therapy according to the guidelines, so we don’t expect significant differences in the influence of antihypertension therapy on outcomes in similar cases. As to the adjusted regression analysis, a limited number of clinical cofounders was taken into account, which is also a limitation of our study. The outcome of the study may be also affected by a bias arising from subjective assessments of the caregivers.

Notwithstanding the foregoing, our study confirms the significance of Pulse Pressure Variability in predicting the functional outcome in AIS, both short and long-term. It brings novel insights concerning the usefulness of six different PPV indices (CV, SV, SD, ARV, DMM and MSC), while most studies focus only on two. The provided analysis is comprehensive and combines a wider range of statistical methods than previous studies. The issues addressed here aids the recent search of the best BPV measures applicable in clinical practice and more importantly, provide the predictors of the functional outcome after ischemic stroke treatment.

In conclusion, elevated PPV during the first 72 h after admission as a result of AIS occurrence is associated with unfavorable outcome at 30 and 90 days, and this association is independent of mean BP levels. All considered PPV measures are reliable stroke outcome predictors. Our recommendation is that clinical trials investigating the benefit of reducing BPV by using antihypertensive medication should monitor also PPV values.

## Data Availability

Data and materials are available on request to the corresponding author.

## References

[1] Qureshi AI (2008). Acute hypertensive response in patients with stroke: pathophysiology and management. Circulation.

[2] Kamieniarz-Mędrygał M, Kaźmierski R (2022). Ambiguities in blood pressure management in acute ischaemic stroke. Neurol. Neurochir. Pol..

[3] Powers WJ, Rabinstein AA, Ackerson T, Adeoye OM, Bambakidis NC, Becker K (2018). 2018 guidelines for the early management of patients with acute ischemic stroke: a guideline for healthcare professionals from the American Heart Association/American Stroke Association. Stroke.

[4] Berge E, Whiteley W, Audebert H, De Marchis GM, Fonseca AC, Padiglioni C (2021). European Stroke Organisation (ESO) guidelines on intravenous thrombolysis for acute ischaemic stroke. Eur. Stroke J..

[5] Levy DE, Brott TG, Haley EC, Marler JR, Sheppard GL, Barsan W (1994). Factors related to intracranial hematoma formation in patients receiving tissue-type plasminogen activator for acute ischemic stroke. Stroke.

[6] National Institute of Neurological Disorders and Stroke rt-PA Stroke Study Group. Tissue plasminogen activator for acute ischemic stroke. *N. Engl.**J. Med*. 1995;333:1581–1587. 10.1056/NEJM199512143332401 7477192

[7] Anderson CS, Huang Y, Lindley RI, Chen X, Arima H, Chen G (2019). Intensive blood pressure reduction with intravenous thrombolysis therapy for acute ischaemic stroke (ENCHANTED): an international, randomised, open-label, blinded-endpoint, phase 3 trial. Lancet.

[8] RIGHT-2 Investigators. Prehospital transdermal glyceryl trinitrate in patients with ultra-acute presumed stroke (RIGHT-2): an ambulance-based, randomised, sham-controlled, blinded, phase 3 trial. *Lancet*. 2019;393:1009–1020. 10.1016/S0140-6736(19)30194-1 PMC6497986 30738649

[9] Vemmos KN, Tsivgoulis G, Spengos K, Zakopoulos N, Synetos A, Manios E (2004). U-shaped relationship between mortality and admission blood pressure in patients with acute stroke. J. Intern. Med..

[10] Leonardi-Bee J, Bath PMW, Phillips SJ, Sandercock PAG (2002). IST Collaborative Group. Blood pressure and clinical outcomes in the International Stroke Trial. Stroke.

[11] Minhas JS, Wang X, Lavados PM, Moullaali TJ, Arima H, Billot L (2019). Blood pressure variability and outcome in acute ischemic and hemorrhagic stroke: a post hoc analysis of the HeadPoST study. J. Hum. Hypertens..

[12] Mistry EA, Mehta T, Mistry A, Arora N, Starosciak AK, De Los Rios La Rosa F (2020). Blood pressure variability and neurologic outcome after endovascular thrombectomy: a secondary analysis of the BEST study. Stroke.

[13] Veloudi P, Sharman JE (2018). Methodological factors affecting quantification of blood pressure variability: a scoping review. J. Hypertens..

[14] Hernandez MF, Chang TI (2023). The need to reduce variability in the study of blood pressure variability. Am. J. Kidney Dis..

[15] Manning LS, Rothwell PM, Potter JF, Robinson TG (2015). Prognostic significance of short-term blood pressure variability in acute stroke: systematic review. Stroke.

[16] Franklin SS, Khan SA, Wong ND, Larson MG, Levy D (1999). Is pulse pressure useful in predicting risk for coronary heart Disease? The Framingham heart study. Circulation.

[17] Vemmos KN, Tsivgoulis G, Spengos K, Manios E, Daffertshofer M, Kotsis V (2004). Pulse pressure in acute stroke is an independent predictor of long-term mortality. Cerebrovasc Dis..

[18] Tsivgoulis G, Spengos K, Zakopoulos N, Manios E, Xinos K, Vassilopoulos D (2005). Twenty four hour pulse pressure predicts long term recurrence in acute stroke patients. J0 Neurol. Neurosurg. Psychiatry..

[19] Lee K-J, Kim BJ, Han M-K, Kim J-T, Cho K-H, Shin D-I (2018). Predictive value of pulse pressure in acute ischemic stroke for future major vascular events. Stroke.

[20] Katsanos AH, Alexandrov AV, Mandava P, Köhrmann M, Soinne L, Barreto AD (2020). Pulse pressure variability is associated with unfavorable outcomes in acute ischaemic stroke patients treated with intravenous thrombolysis. Eur. J. Neurol..

[21] Maïer B, Turc G, Taylor G, Blanc R, Obadia M, Smajda S (2018). Prognostic significance of pulse pressure variability during mechanical thrombectomy in acute ischemic stroke patients. J. Am. Heart Assoc..

[22] Kamieniarz-Mędrygał M, Łukomski T, Kaźmierski R (2021). Short-term outcome after ischemic stroke and 24-h blood pressure variability: association and predictors. Hypertens Res..

[23] Teasdale G, Jennett B (1974). Assessment of coma and impaired consciousness. A practical scale. Lancet.

[24] Członkowska A, Kobayashi A (2013). Expert group of the section of cerebrovascular diseases of the polish neurological society [management of acute stroke - guidelines from the expert group of the section of cerebrovascular diseases of the polish neurological society. update 2013: thrombolysis]. Neurol. Neurochir. Pol..

[25] Yang M, Lu T, Weng B, He Y, Yang H (2020). Association between blood pressure variability and short-term outcome after intra-arterial thrombectomy in acute stroke patients with large-vessel occlusion. Front. Neurol..

[26] Michalak S, Kazmierski R, Hellmann A, Wysocka E, Kocialkowska-Adamczewska D, Wencel-Warot A (2011). Serum paraoxonase/arylesterase activity affects outcome in ischemic stroke patients. Cerebrovasc. Dis..

[27] Adams HP, Leclerc JR, Bluhmki E, Clarke W, Hansen MD, Hacke W (2004). Measuring outcomes as a function of baseline severity of ischemic stroke. Cerebrovasc. Dis..

[28] de Havenon A, Bennett A, Stoddard GJ, Smith G, Wang H, Wold J (2016). Increased blood pressure variability is associated with worse neurologic outcome in acute anterior circulation ischemic stroke. Stroke Res. Treat..

[29] Endo K, Kario K, Koga M, Nakagawara J, Shiokawa Y, Yamagami H (2013). Impact of early blood pressure variability on stroke outcomes after thrombolysis: the SAMURAI rt-PA Registry. Stroke.

[30] Delgado-Mederos R, Ribo M, Rovira A, Rubiera M, Munuera J, Santamarina E (2008). Prognostic significance of blood pressure variability after thrombolysis in acute stroke. Neurology.

[31] Bennett AE, Wilder MJ, McNally JS, Wold JJ, Stoddard GJ, Majersik JJ (2018). Increased blood pressure variability after endovascular thrombectomy for acute stroke is associated with worse clinical outcome. J. Neurointerv. Surg..

[32] Berge E, Cohen G, Lindley RI, Sandercock P, Wardlaw JM, Sandset EC (2015). Effects of blood pressure and blood pressure-lowering treatment during the first 24 hours among patients in the third international stroke trial of thrombolytic treatment for acute ischemic stroke. Stroke.

[33] Martins AI, Sargento-Freitas J, Jesus-Ribeiro J, Correia I, Cardoso L, Gomes JP (2018). Blood pressure variability in acute ischemic stroke: the role of early recanalization. Eur. Neurol..

[34] Agabiti-Rosei E, Mancia G, O’Rourke MF, Roman MJ, Safar ME, Smulyan H (2007). Central blood pressure measurements and antihypertensive therapy: a consensus document. Hypertension.

[35] Millar JA, Lever AF, Burke V (1999). Pulse pressure as a risk factor for cardiovascular events in the MRC Mild Hypertension Trial. J. Hypertens..

[36] Benetos A, Rudnichi A, Safar M, Guize L (1998). Pulse pressure and cardiovascular mortality in normotensive and hypertensive subjects. Hypertension.

[37] Tang S, Yin J, Liu C, Sun M, Lee J, Sun Y (2017). Low pulse pressure after acute ischemic stroke is associated with unfavorable outcomes: the Taiwan Stroke Registry. J. Am. Heart Assoc..

[38] Safar ME (1989). Pulse pressure in essential hypertension: clinical and therapeutical implications. J. Hypertens..

[39] Safar ME (2018). Arterial stiffness as a risk factor for clinical hypertension. Nat. Rev. Cardiol..

[40] Dart AM, Kingwell BA (2001). Pulse pressure—a review of mechanisms and clinical relevance. J. Am. Coll. Cardiol..

[41] Robertson AD, Tessmer CF, Hughson RL (2010). Association between arterial stiffness and cerebrovascular resistance in the elderly. J. Hum. Hypertens..

[42] Liu D, Nie X, Pan Y, Yan H, Pu Y, Wei Y (2021). Adverse outcomes associated with higher mean blood pressure and greater blood pressure variability immediately after successful embolectomy in those with acute ischemic stroke, and the influence of pretreatment collateral circulation status. J. Am. Heart Assoc..

[43] Chang JY, Jeon S-B, Jung C, Gwak DS, Han M-K (2019). Postreperfusion blood pressure variability after endovascular thrombectomy affects outcomes in acute ischemic stroke patients with poor collateral circulation. Front Neurol..

[44] Wang J, Shi X, Ma C, Zheng H, Xiao J, Bian H (2017). Visit-to-visit blood pressure variability is a risk factor for all-cause mortality and cardiovascular disease: a systematic review and meta-analysis. J. Hypertens..

[45] Stevens SL, Wood S, Koshiaris C, Law K, Glasziou P, Stevens RJ (2016). Blood pressure variability and cardiovascular disease: systematic review and meta-analysis. BMJ.

[46] Hassan AKM, Abd-El Rahman H, Mohsen K, Dimitry SR (2017). Impact of in-hospital blood pressure variability on cardiovascular outcomes in patients with acute coronary syndrome. J. Clin. Hypertens (Greenwich)..

[47] Suzuki M, Saito Y, Kitahara H, Saito K, Takahara M, Himi T (2021). Impact of in-hospital blood pressure variability on clinical outcomes in patients with symptomatic peripheral arterial disease. Hypertens Res..

[48] Li J, Yang SC, Moullaali TJ, Chen R, Woodward M, Carcel C (2019). Sex differences in blood pressure after stroke: a systematic review and meta-analysis. J. Hypertens..

[49] Maranon R, Reckelhoff JF (2013). Sex and gender differences in control of blood pressure. Clin. Sci. (Lond)..

[50] Zanchetti A, Facchetti R, Cesana GC, Modena MG, Pirrelli A, Sega R (2005). SIMONA participants Menopause-related blood pressure increase and its relationship to age and body mass index: the SIMONA epidemiological study. J. Hypertens..

[51] Feigin VL, Norrving B, Mensah GA (2017). Global burden of stroke. Circ. Res..

[52] Roquer J, Campello AR, Gomis M (2003). Sex differences in first-ever acute stroke. Stroke.

